# First demonstration of coherent resonant backward diffraction radiation for a quasi-monochromatic terahertz-light source

**DOI:** 10.1038/s41598-020-64426-1

**Published:** 2020-05-05

**Authors:** Norihiro Sei, Toshiharu Takahashi

**Affiliations:** 1 Research Institute for Measurement and Analytical Instrumentation, National Institute of Advanced Industrial Science and Technology, Umezono 1-1-1, Tsukuba, Ibaraki 305-8568 Japan; 2 Institute for Integrated Radiation and Nuclear Science, Kyoto University, 2 Asashiro-nishi, Kumatori, Osaka 590-0494 Japan

**Keywords:** Terahertz optics, Optoelectronic devices and components

## Abstract

We proposed coherent resonant backward diffraction radiation (CRBDR), which generates wavelength-tunable quasi-monochromatic lights using a compact diffractor assembly in an accelerator facility of high-energy electron beams, as a unique intense terahertz (THz) light source. Superimposing the coherent backward diffracted radiation emitted by periodically arranged hollow diffractors, it is possible to amplify the frequency components satisfying a resonant condition, and make the radiation monochromatic. We demonstrated the CRBDR using the L-band linac at the Institute for Integrated Radiation and Nuclear Science at Kyoto University. It was observed that the coherent backward diffraction radiation was amplified more than three times at a frequency which was the fundamental resonant frequency in the CRBDR theory. Moreover, the number of diffractors at the saturation of the radiation power was consistent with the number estimated from the electron distribution in a bunch. The experimental results show that the CRBDR is useful as a quasi-monochromatic light source in the THz band.

## Introduction

In the terahertz (THz) band, there are various low-frequency excitations such as rotation modes of macromolecules and superconducting gap modes^1,2^. The THz band can be used as a fingerprint region for useful polymers^3^, and several applied technologies, such as noninvasive imaging and substance identification^4,5^, have been developed with broadband THz lights. Recently, applications of high-power monochromatic THz lights have also been activated. For example, it has been reported that the fibrous structure of a peptide can be dissociated using a monochromatic THz light without the influence of the thermal effect^6^. Although the demand for high-power wavelength-tunable lights are expected to increase in the THz band, there are not many THz-light sources that can be used as excitation sources. Therefore, accelerator-based THz-light sources which can be used for any application have been developed^7–9^. The radiation principle mainly used in the development of the accelerator-based THz-light sources is a free-electron laser (FEL)^10,11^. Several FEL devices with high peak and average powers have been developed and contributed to applied research in the THz band^12,13^. However, it is difficult to produce a compact FEL device because the FEL requires an undulator with many periods and an optical resonator. An appealing factor of accelerator facilities is that they can supply multiple quantum sources simultaneously. To facilitate simultaneous usage of THz lights with other accelerator-based quantum sources, the development of a tunable high-power light source in the THz band, by inserting a small device into the existing electron beam trajectory, is desired.

Therefore, we have addressed the development of a new light source, which superimposes multiple coherent radiations and makes them monochromatic, using temporal coherence^14^. The coherent radiation is a phenomenon that radiation from individual electrons in an electron bunch interferes and the radiation intensity increases nonlinearly when the electrons emit light having wavelengths longer than the bunch length^10^. Studies on coherent undulator radiation (CUR) have been reported as the monochromatic THz-light source superimposing multiple coherent radiations^15,16^. However, the undulator radiation emitted forward on the electron beam trajectory is shifted to the short wavelength side due to the Lorentz contraction. A long undulator is necessary to obtain high-power THz lights, and it is difficult to make the CUR compact. To reduce the size of the light source, it is suitable to use coherent radiation, emitted backward, on the electron beam trajectory. Smith-Purcell backward wave oscillator using a metal diffraction grating has been developed as a powerful monochromatic light source in the THz band^17,18^. However, a slow wave circuit is formed by a spatially limited waveguide in the Smith-Purcell backward wave oscillator, so that the energy of the electrons must be low. It is difficult to use in combination with other accelerator-based quantum sources such as undulator radiation. Diffraction radiation^19^, which is generated by a charged relativistic particle going through an aperture in a conductor, is emitted both, in front and behind the conductor. The diffraction radiation can generate lights with shorter wavelengths as the energy of the charged particle increases^20,21^. Unlike transition radiation, it does not attenuate the charged particle beam in the conductor. Moreover, unlike Smith-Purcell backward wave oscillator, it can be considered to propagate in free space without interacting vacuum chambers surrounded the electron beam. By periodically arranging hollow metal diffractors and passing a relativistic electron bunch through the holes of the diffractors, coherent diffraction radiation is emitted backward from each diffractor. A high-power quasi-monochromatic beam in the THz band can be expected by superimposing multiple coherent diffraction radiations. In this article, we provided a basic theory for this coherent resonant backward diffraction radiation (CRBDR), and then report the results of the demonstration experiments conducted at the Institute for Integrated Radiation and Nuclear Science at Kyoto University (KURNS-LINAC).

## Methods

### Coherent backward diffraction radiation

Backward transition radiation consists electromagnetic waves that are emitted to the vacuum side due to temporal variations of an electric dipole, which is composed of a charged particle passing through a conductor and a mirror image charge appearing in the conductor. In this study, an electron is considered as the charged particle. When the electron enters the conductor surface perpendicular from the vacuum, the intensity of the backward transition radiation emitted per unit frequency and solid angle, is given in a frequency region lower than the plasma frequency, according to the following approximated expression^22^:

1 $$\frac{d{I}_{TR}}{d\varOmega d\omega }=\frac{{e}^{2}{\beta }^{2}}{4{\pi }^{3}{\varepsilon }_{0}c}{\left[\frac{\sin \theta }{1-{\beta }^{2}{\cos }^{2}\theta }\right]}^{2},$$

where *e*, *c*, *θ*, and *β* is the electron charge, speed of light, polar observation angle, and the ratio of the electron speed to *c*, respectively. Because a mirror image charge also appears in a conductor when an electron passes through the aperture in the conductor, the backward diffraction radiation is generated from the aperture boundary to the vacuum when the electron is closest to the aperture boundary. By making the diameter of the aperture zero, the backward diffraction radiation becomes to match the backward transition radiation. Therefore, the intensity of the backward diffraction radiation can be described with the intensity of the backward transition radiation, *I*_*TR*_. When a relativistic electron passes through the centre of a hole with a diameter *D* perpendicularly in a thin conductor, the intensity of the backward diffraction radiation emitted per unit frequency and solid angle is given with the Bessel function of the first kind *J*_0_ and modified Bessel function of the second kind *K*_1_ by

2 $$\frac{d{I}_{DR}}{d\varOmega d\omega }=\frac{d{I}_{TR}}{d\varOmega d\omega }{\left[\frac{a}{\beta \gamma }{J}_{0}(a\sin \theta ){K}_{1}\left(\frac{a}{\beta \gamma }\right)\right]}^{2}$$

3 $$a=\frac{D\omega }{2c},$$

where *γ* is the electron energy in units of its rest mass^20^. The conductor is assumed to be an infinite plane in these equations. The diameter of the conductor used in diffraction radiation experiments is usually less than 100 mm. According to^23^, Eq. (2) is generally effective in the frequency region of 0.1 THz or higher.

The electron beam, accelerated by high-frequency electric field in a linac, forms spatially isolated electron bunches. For a wavelength region longer than the bunch length, the radiations emitted by the individual electrons in the bunch superimpose coherently, and the intensity of the entire radiation becomes nonlinear with respect to the number of electrons in the bunch, *N*_*e*_. Such radiation is called coherent radiation, and the intensity spectral-angular distribution of coherent backward diffraction radiation (CBDR), which is a type of coherent radiation, can be expressed as follows using Eq. (2) ^8,24^:

4 $$\frac{d{I}_{CBDR}}{d\varOmega d\omega }=\frac{d{I}_{DR}}{d\varOmega d\omega }{N}_{e}[1+({N}_{e}-1)f(\omega )]$$

5 $$f(\omega )={|{\int }_{-\infty }^{\infty }\exp \left(i\frac{\omega }{c}z\right)S(z)dz|}^{2}$$

where *z* is the direction of the electron-beam motion, and *S*(*z*) is the normalised density distribution function of the electron in the bunch. The form factor *f*(*ω*) has a value from 0 to 1 and approaches 1 for wavelengths longer than the bunch length. Then, depending on the bunch length, the CBDR becomes an intense light source from the millimetre-wave band to the THz band.

### Theory of coherent resonant backward diffraction radiation

**Figure 1:**
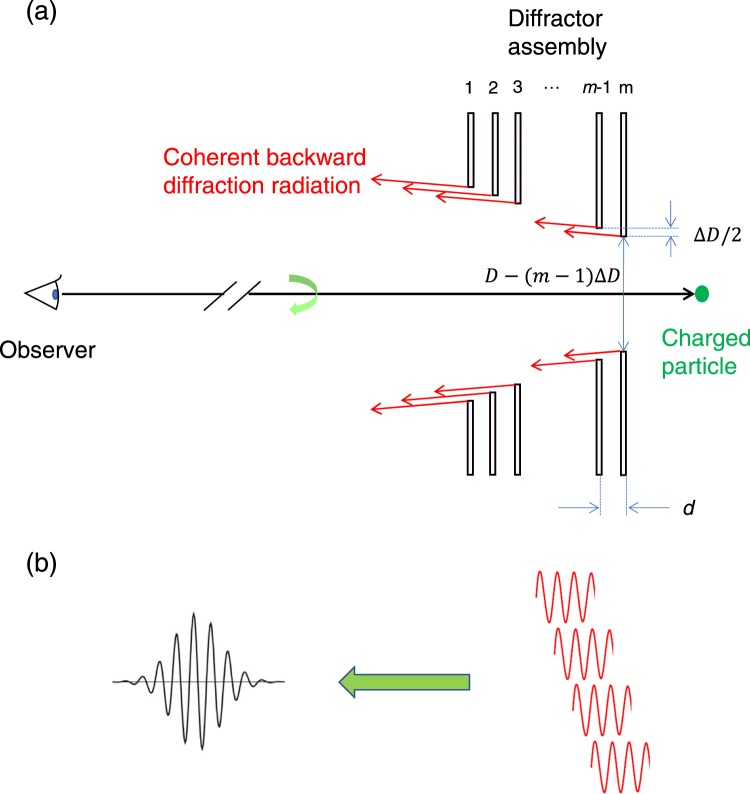
(**a**) Schematic layout of the coherent backward diffraction radiation with periodic diffractors, and (**b**) schematic diagram of the wave packet of the coherent resonant backward diffraction radiation on which the coherent backward diffraction radiation emitted from each diffractor is superimposed.

As shown in Eq. (4), CBDR is emitted in the wide frequency region, and the spectral-angular distribution of the intensity is insufficient to use in high-resolution spectroscopic measurements. To obtain quasi-monochromatic radiation by increasing the power, it is effective to cause interference in the radiation by a periodic structure. In fact, accelerator-based light sources with a periodic structure such as CUR^16^, coherent Smith-Purcell radiation^25^, and coherent Cherenkov radiation using a waveguide^26^ have already been developed as quasi-monochromatic light sources in the THz band. Resonant transition radiation, which narrows the line width of the radiation by superimposing forward transition radiation generated from multilayer thin films, has also been studied for a long time^27,28^, though it is in the X-ray band. By arranging the conductive diffractor periodically and observing radiation under the Fraunhofer condition as shown in Fig. 1(a), the electric field of the CBDR emitted from each diffractor can be superimposed as shown in Fig. 1(b). The intensity of such radiation, which we call CRBDR^14^, increases nonlinearly at wavelengths where the radiation phase emitted from each diffractor is matched. Despite the simple structure, CRBDR is expected to be an accelerator-based quasi-monochromatic light source in the THz band. When a temporally uniform relativistic electron beam passes through the centres of circular holes bored in *m* conductive diffractors arranged in parallel, the electric field measured by a distant observer, *E*_*Ob*_(*θ*, *ω*), is given by

6 $${E}_{Ob}(\theta ,\omega )=\mathop{\sum }\limits_{j=1}^{m}{E}_{j}{\prime} (\theta ,\omega ),$$

where *E*′_*j*_(*θ*, *ω*) is the electric field that is CBDR generated by the *j*th diffractor and reaches the observation point after passing through the upstream diffractors, and *θ* is the elevation angle in which the incident direction of electron beam is positive. The integral of |*E*_1_(*θ*, *ω*)|^2^ with respect to the elevation angle and the angular frequency is proportional to *I*_*CBDR*_. According to the theory of resonant transition radiation^27^, it is assumed that the influence of diffractors between a radiated diffractor and the observer on the electric field is divided into a phase factor ϕ and a loss factor *α*. Because the electric field generated by each diffractor is equal, *E*′_*j*_(*θ*, *ω*) can be expressed with *E*_1_ (*θ*, *ω*) by the following equation:

7 $${E}_{j}{\prime} (\theta ,\omega )={E}_{1}(\theta ,\omega ){e}^{-i{\phi }_{j}}{e}^{-{\alpha }_{j}/2}$$

According to the Smith-Purcell radiation analogy^29^, the phase can be expressed with a period of the diffractors *d* by the following equations:

8 $${\phi }_{j}=(j-1)\Delta \phi ,$$

9 $$\Delta \phi =\frac{\omega }{c}d\left(\frac{1}{\beta }+\,\cos \,\theta \right)\approx \frac{2d\omega }{c}.$$

when the electron energy is sufficiently relativistic (*γ*≫ 1), a relationship that the spectral-angular distribution of the intensity of the CBDR has a maximum at *θ* = 1/*γ* is used in the approximation of Eq. (9). It is found that the CRBDR resonates at frequencies where the phase difference, Δ*ϕ*, given by Eq. (9) equals to a positive integer multiple of 2π. That is, the frequency, *f*, in the resonant condition is given with a positive integer, *n*, by

10 $$f=\frac{\omega }{2\pi }=\frac{c}{2d}n$$

The loss factor, *α*, includes two components of loss: due to the transportation system and diffractors, which are expressed as *α*_*T*_ and *α*_*d*_, respectively. The loss factor *α*_*d*_ appears when the number of diffractors is more than 2, and it can be assumed that the contribution of each diffractor to *α*_*d*_ is equal. Thus, the following relationship holds for the *j*th diffractor (*j* ≥ 2):

11 $${e}^{-\frac{{\alpha }_{j}}{2}}\approx {e}^{-\frac{{\alpha }_{T}}{2}-(j-1)\frac{{\alpha }_{d}}{2}}.$$

**Figure 2:**
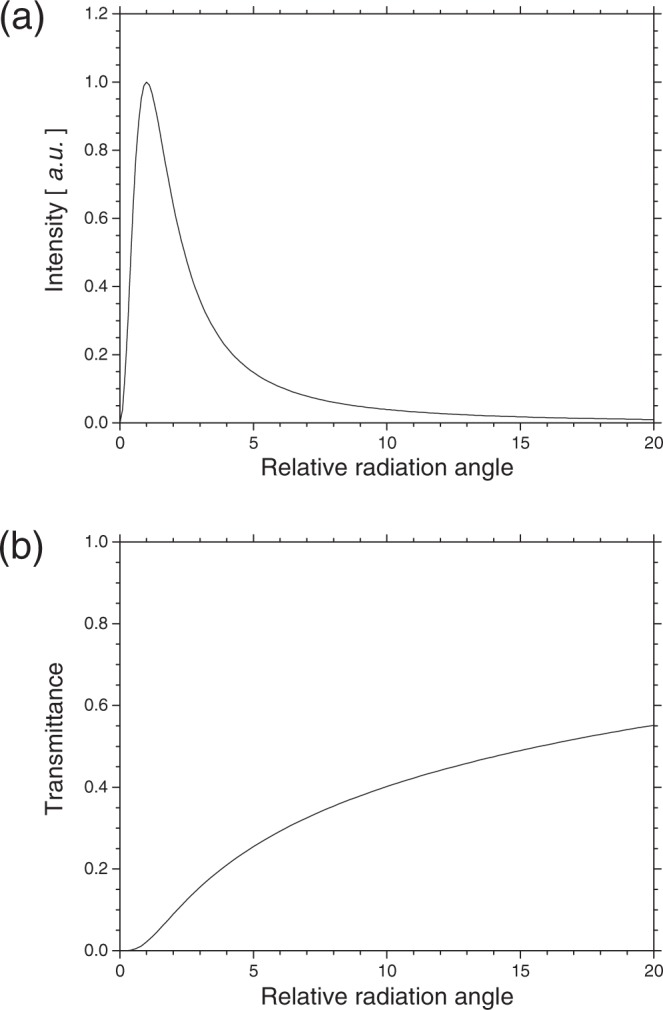
(**a**) Dependence of the intensity distribution of the backward transition radiation on the radiation angle in unit of 1/*γ*, and (**b**) the ratio of the intensity of the backward transition radiation emitted up to a certain angle to the total energy at *γ* = 82.

The transmittance efficiency with which radiation emitted from a diffractor passes through the adjacent diffractor is important for the CRBDR. The diffraction radiation is emitted with an expansion beyond the hole diameter of the diffractor. Because the diffraction radiation is shielded by the upstream diffractors when the hole diameters of all diffractors are equal, multiple radiations cannot be effectively superimposed. Therefore, the structure of the diffractor assembly is adopted in which the hole diameter of the diffractor is gradually reduced along the direction of the electron-beam motion to suppress the shielding effect of the upstream diffractors. As shown in Eq. (4), the angular dependence of the spatial distribution of the backward diffraction radiation is mainly dominated by that of the backward transition radiation. Figure 2(a) shows the dependence of the intensity distribution of the backward transition radiation on the radiation angle normalised by 1/*γ*, which is calculated with Eq. (1). Although the intensity distribution has a maximum when the radiation angle is 1/*γ*, decrement of the intensity distribution is gradual in a region of the larger radiation angle. The ratio of the intensity of the backward transition radiation emitted up to a certain angle to the total intensity of the backward transition radiation at *γ* = 82, is shown in Fig. 2(b). It is noted that transporting the transition radiation with a large solid angle is necessary to obtain a high-power light source. When an elevation angle caused by the difference, Δ*D*, between the diameters of adjacent diffractors, *φ* = Δ*D*/2*d*, is smaller than the observation angle allowed by the transportation system of the CBDR, *θ*_*O*_, an effect of interference generated by the diffractor assembly in the observed radiation is reduced due to the diffraction loss of the upstream diffractors. Therefore, in the CRBDR, Δ*D* should be selected so that *φ* is larger than *θ*_*O*_. However, the large Δ*D* significantly reduces the high-frequency components of the CRBDR as shown in Eq. (4). To obtain high-power quasi-monochromatic THz lights by the CRBDR, it is preferable to increase the electron energy. In addition, if the hole centre of each diffractor is randomly shifted from the electron-beam orbit, the shifted diffractor shields radiation emitted from the downstream diffractors. The installation accuracy of the diffractors is a significant matter for the CRBDR.

The electron beam of an actual linac is not uniform temporally but has a bunching structure. The envelope of the electric field of the CBDR can be expressed as *S*(*t*) converting the electron density distribution in Eq. (5) into a function of time. When the centre of the electron bunch passes through the hole of the first diffractor, the time *t* is set to zero. The envelope of the observed electric field of the CBDR emitted by the *j*th diffractor, *E*′_*j*_ (*θ*, *ω*), is modified using the electron-density distribution as the following equation:

12 $${E}_{j}{\prime} (\theta ,\omega )\approx {E}_{1}(\theta ,\omega ){e}^{-i{\phi }_{j}}{e}^{-\frac{{\alpha }_{T}}{2}-(j-1)\frac{{\alpha }_{d}}{2}}S\left(t+2(j-1)\frac{d}{c}-\tau \right),$$

where *τ* is the time it takes the radiation emitted from the first diffractor to reach the observer. The maximum power of the CRBDR in the resonant condition is calculated from the sum of the electric fields, Σ*E*′_*j*_ (*θ*, *ω*). As Eq. (12) indicates, the electron-bunch length should be longer than the period of the diffractors to superimpose the CBDR emitted by each diffractor effectively. When the electron-bunch length is sufficiently longer than the length of the diffractor assembly, *S*(*t* + 2*md/c*) can be approximated to equal to *S*(*t*), and the bunch structure of the electron beam is negligible in this radiation, as well as the resonant transition radiation^27^. However, the power of the coherent radiation becomes strikingly lower at wavelengths shorter than the bunch length^24^. The power of multiple diffraction radiations with a periodic structure and the power of the coherent radiation generated in a short electron bunch are in a trade-off relationship. An amplification factor, which is the ratio of the radiation intensity emitted from a diffractor assembly to that emitted from a diffractor, is a good measure of the performance of the diffractor assembly. Table 1 shows the maximum values of the amplification factors of the CRBDR power emitted by the diffractor assembly and form factors at the frequency of the fundamental harmonic, for some diffractor periods normalised by the bunch length. The loss factor *α* is assumed to be negligible in these estimations. Because the maximum amplification factor normalised with the CBDR power of one diffractor depends on *S*(*t*), a triangular distribution, which is observed in a high charge linac^30^, and a Gaussian distribution are considered as the bunch structure. The bunch length, *σ*_*l*_, is the standard deviation for the Gaussian distribution and the half width at half maximum for the triangular distribution. Because the form factor for the triangular distribution is a function of frequency oscillating with a period of 2*c*/*σ*_*l*_, values calculated using the envelope of the function are shown in Table 1. It is known that such an envelope can explain the form factor obtained in the experiments^30^. As shown in Table 1, the maximum amplification factor for the diffractor assembly with 5 diffractors is more than 10 at the diffractor period of approximately 0.5*σ*_*l*_. It is difficult to obtain sufficient CRBDR power due to the extremely low form factor for the Gaussian distribution, which is an ideal bunch structure. However, in some linac facilities with the complex structure of the electron bunch^30,31^, the observed spectrum of coherent transition radiation was maximised with wavelengths shorter than the bunch length. In such facilities, it is expected to generate quasi-monochromatic CRBDR with an amplification factor of more than 10 at the resonant frequency using a diffractor assembly with the diffractor period of approximately 0.5*σ*_*l*_.

**Table 1:** Maximum relative amplification factors and form factors for some diffractor periods normalised by the bunch length. The bunch structure is assumed to be Gaussian or triangle distribution.

Period of diffractor to the bunch length	Maximum relative amplification factor				Form factor for fundamental harmonic	
	Gaussian		Triangle		Gaussian	Triangle
	5 sheets	10 sheets	5 sheets	10 sheets		
0.1	24.0	86.3	23.7	82.6	<10^−10^	1.0 × 10^−6^
0.2	21.6	63.8	20.8	57.7	<10^−10^	1.6 × 10^−5^
0.5	13.9	31.6	12.4	27.4	<10^−10^	6.4 × 10^−4^
1.0	8.1	16.9	7.0	14.5	5.2 × 10^−5^	1.0 × 10^−2^

### Experimental setup at KURNS-LINAC

The demonstration experiments of the CRBDR have been performed with an L-band electron linac at KURNS-LINAC. Various types of coherent radiations have been studied in this facility^31–34^, and a THz-band beamline that can supply coherent transition radiation for spectroscopic experiments has been constructed^35^. Because a large diffractor assembly with a volume of 10 cm^3^ or more can be installed in the aluminium vacuum chamber which is used for generation of the coherent transition radiation, the CBDR can generate at frequencies less than 0.1 THz.

**Figure 3:**
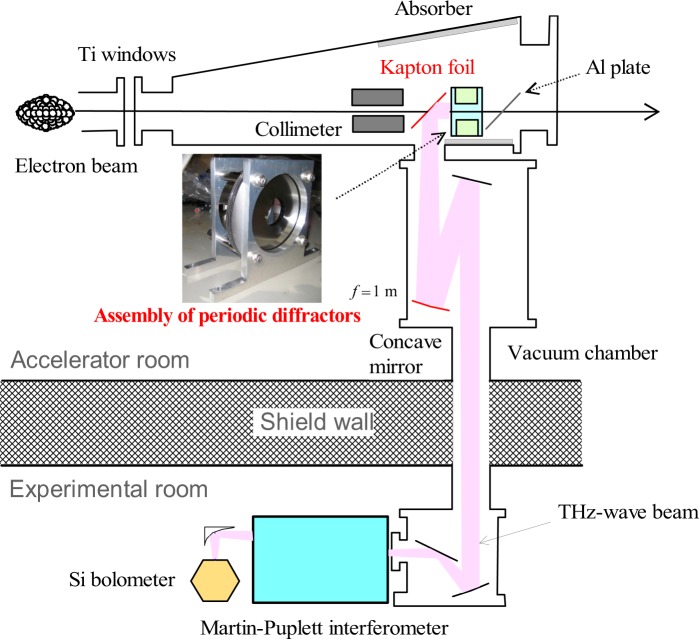
Schematic layout of the CRBDR in KURNS-LINAC.

A photograph of the diffractor assembly used in the experiments is shown in Fig. 3. The diffractor was a steel disk with a diameter of 107 mm and a thickness of 0.1 mm.To reduce a phase error of the CRBDR due to the thickness of the diffractor less than 5%, the diffractor period was set to more than 1.8 mm. The minimum hole diameter of the diffractor was 30 mm, and this diffractor was located at the most downstream. When one diffractor was added to the diffractor assembly upstream, the hole diameter increased by 0.2 mm. Four guide shafts (diameter 6 mm), arranged at an equal angle to circumscribe the circumference of the diffractor, fixed the diffractors. The installation error of the diffractors in the plane perpendicular to the electron beam was within 0.1 mm. The diffractor period could be changed discretely by adjusting a thickness of the acrylic washers passed through the guide shaft. The guide shaft was wrapped with a Kapton seal, and each diffractor was insulated. Two T-shaped aluminium plates fixed the guide shafts. This aluminium plate had a hole with a diameter of 100 mm, and the electron beam passed through the centre of the hole. The distance between the aluminium plates could be increased up to 90 mm.

The schematic layout of the CRBDR experiment with the diffractor assembly is shown in Fig. 3. Although the electron beam size was approximately 3 mm, an aluminium collimator with an inner diameter of 17.5 mm was set upstream of the diffractor assembly to prevent the electron beam from irradiating the diffractors. A thin Kapton film with an effective diameter of 82 mm was installed between the aluminium collimator and the diffractor assembly at an angle of 45 degrees to the electron beam. The CRBDR emitted from the diffractor assembly was reflected by the thin Kapton film and transported to the experimental room. Although the reflectance of the thin Kapton film was approximately 10% in the THz band, it did not generate the coherent transition radiation and the background was reduced. A thin aluminium film was installed downstream of the diffractor assembly at an angle of 45 degrees to the electron beam. It prevented coherent transition radiation, which generated when the electron beam was exited from the aluminium vacuum chamber, from entering the transport pass of the CRBDR beam. Millimetre-wave absorbers (E&C Engineering Inc., ECCOSORB AN72) were set on the vertical surface of the aluminium vacuum chamber to remove the coherent transition radiation. However, a part of the coherent forward diffraction radiation generated at the exit of the aluminium collimator was transported with the CRBDR beam as the background. The CRBDR beam reflected by the thin Kapton film was converted into a parallel beam using a concave mirror with a focal distance of 1.0 m and a diameter of 0.13 m, and transported in vacuum to the experimental room.

## Results

**Figure 4:**
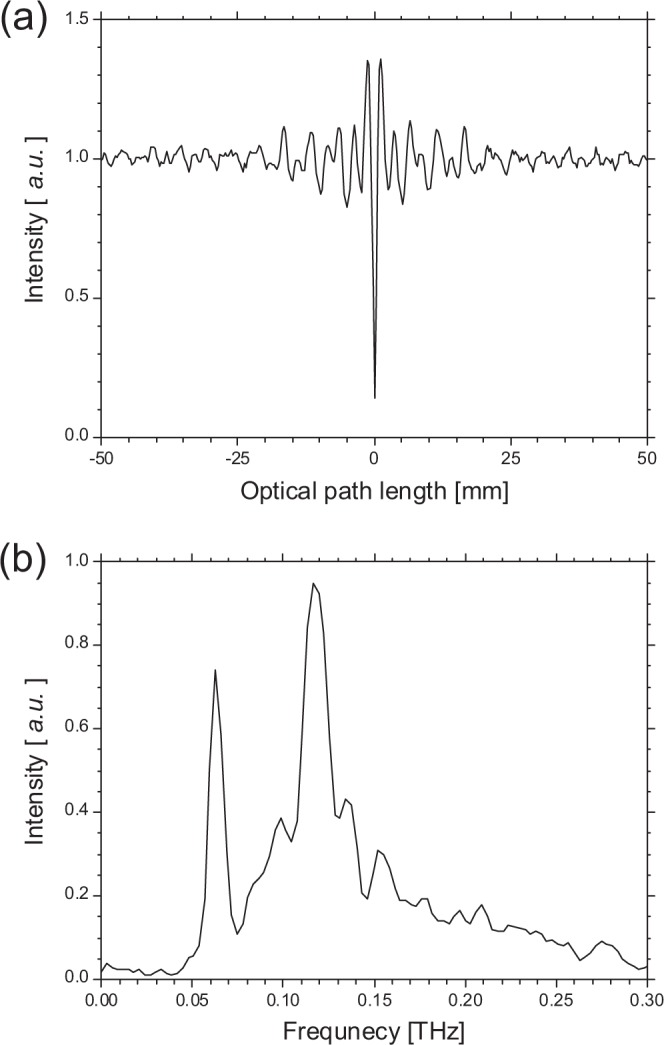
(**a**) Measured interferogram and (**b**) spectrum of the CBDR with one diffractor.

**Figure 5:**
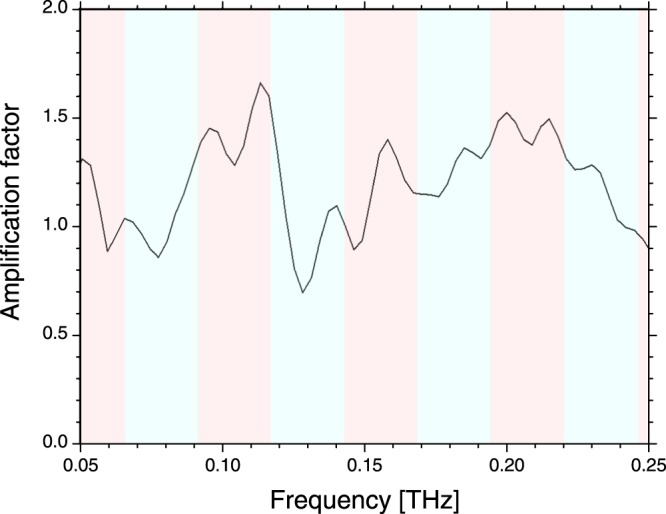
Measured spectrum of the amplification factor for the CRBDR composed of three diffractors with the period of 2.9 mm.

To reduce the diffraction loss, the electron energy was set to 42 MeV, which was the upper limit of the facility. Therefore, the CRBDR beam with the condition of *θ*_*O*_/*γ* < 4 could be observed in this transport system. The spectrum of the CRBDR beam was measured using a Martin-Puplett-type interferometer and a Si bolometer^35^. Because the accelerating frequency was 1.3 GHz, the interval between the micropulses of the electron beam was 770 ps. The macropulse duration and repetition were 10 ns and 30 Hz, respectively. The charge in a micropulse ranged from 0.3 to 0.4 nC. Figure 4 shows a measured interferogram and spectrum of the CBDR with one diffractor. The observed interferogram indicates that the coherent area in the direction of the electron motion was approximately 18 mm. The spectrum had a maximum at the frequency of 0.12 THz, and intense radiation was observed in a frequency region of 0.06 to 0.25 THz. The observed spectrum had a complex structure, and it showed that the electron-bunch structure was not simple Gaussian. There were several periodic peaks in the observed spectrum. This was a typical spectrum of the coherent radiation emitted from an electron bunch with a triangular distribution, and such spectra were observed in L-band linac facilities whose electron bunches were long and high charge^30^. Considering the coherent area indicated by the interferogram, the electron-bunch structure would be assumed to have a triangular distribution with a full width at half maximum of approximately 9 mm. The diffractor period is a significant parameter for effectively measuring the interference effect of the multiple CBDR. When the intensity of the CBDR is low in the attenuation phase, the intensity of the multiple CBDR becomes lower in the attenuation phase and it is difficult to accurately evaluate the amplification factor. The diffractor period should be adjusted so that frequency regions with high and low intensity have an attenuation phase and an amplification phase, respectively. Therefore, the diffractor period was selected so that the amplification phase was in the frequency region of 0.08–0.10 THz and the attenuation phase was in the frequency region of 0.12–0.14 THz. First, the diffractor period was set to 2.9 mm and the fundamental frequency evaluated from Eq. (10) was evaluated 52 GHz. Figure 5 shows the observed spectrum of the amplification factor for the CRBDR composed of three diffractors, whose overall length was shorter than the full width at half maximum of the bunch length. The attenuation and amplification phases calculated with the diffractor period of 2.9 mm are shown as light blue and light pink areas in this figure. Although significant amplification of the CRBDR could not be confirmed due to the low power at the fundamental frequency, the amplification factor had a maximum value exceeding 1.6 at the frequency of 0.11 THz which corresponded to the second harmonics frequency. Moreover, it is noted that the observed spectrum had a period of 52 GHz up to a frequency of 0.16 THz, which corresponded to the third harmonic frequency. Because the elevation angle, *φ*, was 34.5 mrad and was lower than the observation angle *θ*_*O*_ (50 mrad), the sufficient amplification factor could not be obtained with the condition of *d* = 2.9 mm.

**Figure 6:**
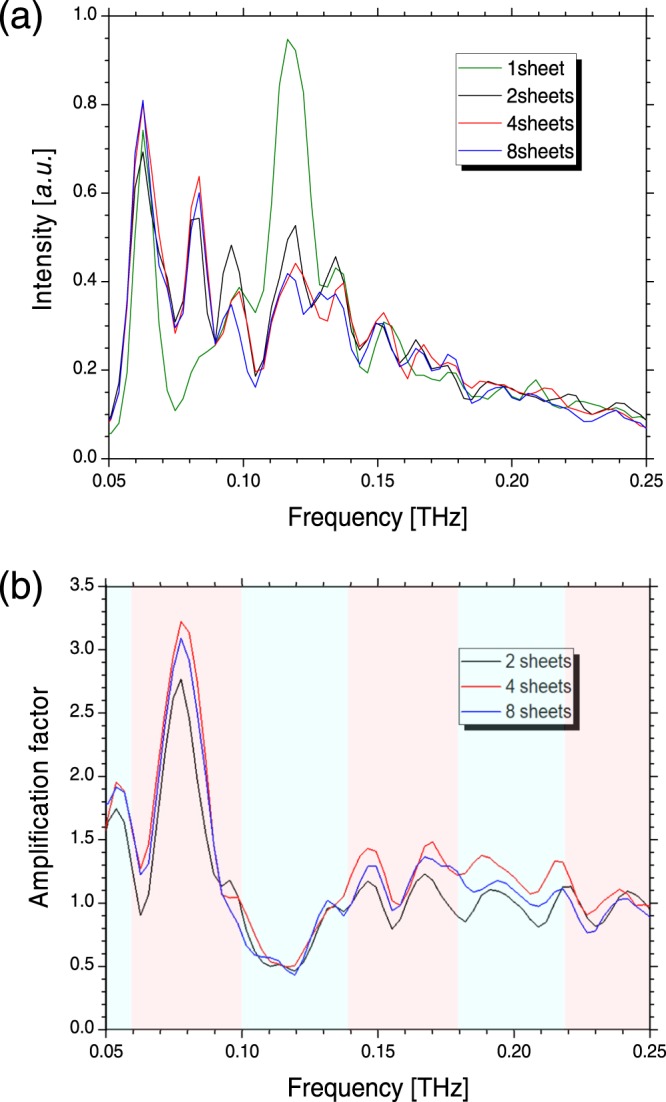
(**a**) Measured spectra of the CRBDR and (**b**) calculated spectra of amplification factor with the period of 1.9 mm. Green, black, red, and blue solid lines are the spectra for 1, 2, 4, and 8 diffractors, respectively.

**Figure 7:**
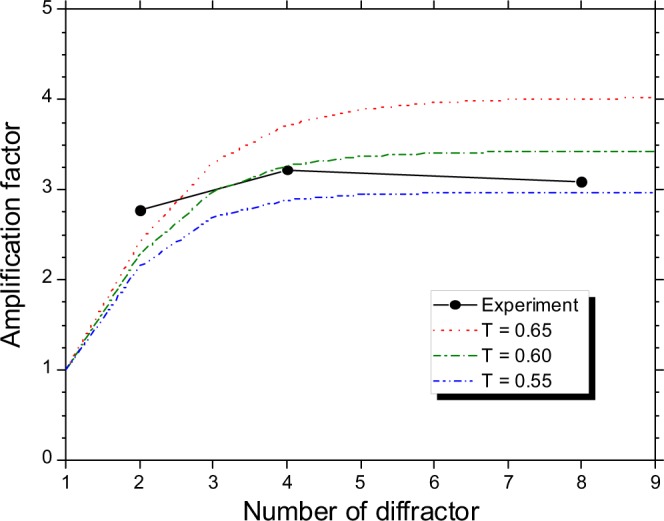
Relationship between the amplification factor and the number of the diffractors at the fundamental resonant frequency. Solid circles are the measured data. The dotted line, the one-dot chain line, and the two-dot chain line represent the amplification factors at the transmittances of 0.65, 0.60, and 0.55, respectively.

Therefore, the diffractor period was set to 1.9 mm, and the fundamental frequency satisfying the resonant condition was 79 GHz. The elevation angle for this diffractor period was 52.6 mrad and was larger than the observation angle *θ*_*O*_. Figure 6(a) shows spectra of the CRBDR with the number of the diffractors of 1, 2, 4, and 8. Moreover, Fig. 6(b) shows spectra of the amplification factor when the number of the diffractors were 2, 4, and 8. The attenuation and amplification phases calculated with the diffractor period of 1.9 mm are indicated as light blue and light pink areas in Fig. 6(b). It is noted that the amplification factor exceeded 3 at the fundamental frequency of 0.08 THz and was halved at 0.12 THz corresponding to the attenuation phase. When the number of the diffractors was 2, amplification due to the superposition of CBDR was not sufficient at the fundamental frequency and a full width at half maximum for the fundamental could not defined as shown in Fig. 6(a). However, the spectral peak at the fundamental frequency narrowed as the number of diffractors increased. The full width at half maximum for the fundamental was evaluated to be 8.1 GHz when the number of diffractors was 4. The narrowing of the spectrum indicates that the electric field from each diffractor resonated. The amplification factor increased to approximately 1.5 times at around the resonance frequency of the second harmonic (0.16 THz). However, the periodic structure became unclear in the frequencies which was higher than those of the second harmonic. It is considered that the error of the diffractor period causes the decrease of the amplification factor in the higher frequency region. According to a calculation using Eq. (12), when there is a standard deviation of 0.1 mm in the diffractor period of 2.9 mm, the amplification factor at the frequency of the second harmonic is reduced by half compared to the amplification factor without the error. Moreover, the measured spectra of the CRBDR in the low frequency region (<0.06 THz) were inaccurate due to low power. The amplification factor was maximised when the number of diffractors was four. The measured relationship between the amplification factor and the number of the diffractors is plotted in Fig. 7. This figure also shows the calculated relationship for some transmittance per diffractor exp(−*α*_*d*_/2) when the bunch structure had a triangular distribution with a full width at half maximum of 9 mm, which was in accord with the measurement data. It is noted that the transmittance was approximately 0.6. To increase the amplification factor, the transmittance should be close to 1, and it was necessary to improve the installation accuracy of each diffractor. It was demonstrated that the theory of the CRBDR was consistent with the experimental results for the resonance frequency and the number of the diffractors saturating the radiation power.

## Discussion

The principle of the CRBDR was proposed as a means of obtaining a quasi-monochromatic THz-light source using a relativistic electron beam and a compact light emitting device. Using a diffractor assembly composed of steel diffractors with a diameter of 107 mm and thickness of 0.1 mm, spectra of the CRBDR were measured with the diffractor periods of 2.9 and 1.9 mm. It was demonstrated that the CRBDR was amplified at the resonance frequencies predicted by the CRBDR theory and the number of diffractors whose CRBDR power was saturated was dependent on the electron distribution in a bunch; then, we observed the formation of CRBDR for the first time. The amplification factor exceeded 3 at the resonance frequency of the fundamental harmonic. In these experiments, because it was difficult to maintain parallelism of large-diameter metallic films with a thickness of less than 0.1 mm, the diffractor period was relatively large. Using thin wafers with aluminium deposited on the surface were used as the diffractors^36^, it is possible to generate the CRBDR with a shorter diffraction period. By reducing the installation error of the diffractor to improve the transmittance up to 0.8, and using a diffractor assembly composed of 8 diffractors with the period of 1 mm, the amplification factor is expected to be approximately 10 at the resonance frequency of the fundamental harmonic.

In the KURNS-LINAC, the frequency suitable for generation of CRBDR is low because the bunch length is long. However, using a high-quality electron beam in an advanced accelerator^37^, a diffractor assembly with a larger number of diffractors, and smaller hole diameter is available for the CRBDR experiments, and it is possible to develop a high-power light source with a high monochromaticity at frequencies approximately 1 THz. Moreover, by connecting the diffractors with springs or piezo elements to adjust the diffractor period, the CRBDR becomes a wavelength-tunable light source in the THz band. Because the compact diffractor assembly can be installed without losing the electron beam, it is possible to use the CRBDR in combination with other accelerator-based light sources. The CRBDR will be a predominant tunable light source in the THz band.
